# A Pipeline for the Implementation and Visualization of Explainable Machine Learning for Medical Imaging Using Radiomics Features

**DOI:** 10.3390/s22145205

**Published:** 2022-07-12

**Authors:** Cameron Severn, Krithika Suresh, Carsten Görg, Yoon Seong Choi, Rajan Jain, Debashis Ghosh

**Affiliations:** 1 Department of Biostatistics and Informatics, University of Colorado, Aurora, CO 80045, USA; cameron.severn@gmail.com (C.S.); carsten.goerg@cuanschutz.edu (C.G.); debashis.ghosh@cuanschutz.edu (D.G.); 2 Department of Radiology, Yonsei University College of Medicine, Seoul 03722, Korea; yoonseong.choi07@gmail.com; 3 Department of Radiology, NYU Grossman School of Medicine, New York, NY 10016, USA; rajan.jain@nyulangone.org; 4 Department of Neurosurgery, NYU Grossman School of Medicine, New York, NY 10016, USA

**Keywords:** explainable machine learning, medical imaging, information visualization, radiomics

## Abstract

Machine learning (ML) models have been shown to predict the presence of clinical factors from medical imaging with remarkable accuracy. However, these complex models can be difficult to interpret and are often criticized as “black boxes”. Prediction models that provide no insight into how their predictions are obtained are difficult to trust for making important clinical decisions, such as medical diagnoses or treatment. Explainable machine learning (XML) methods, such as Shapley values, have made it possible to explain the behavior of ML algorithms and to identify which predictors contribute most to a prediction. Incorporating XML methods into medical software tools has the potential to increase trust in ML-powered predictions and aid physicians in making medical decisions. Specifically, in the field of medical imaging analysis the most used methods for explaining deep learning-based model predictions are saliency maps that highlight important areas of an image. However, they do not provide a straightforward interpretation of which qualities of an image area are important. Here, we describe a novel pipeline for XML imaging that uses radiomics data and Shapley values as tools to explain outcome predictions from complex prediction models built with medical imaging with well-defined predictors. We present a visualization of XML imaging results in a clinician-focused dashboard that can be generalized to various settings. We demonstrate the use of this workflow for developing and explaining a prediction model using MRI data from glioma patients to predict a genetic mutation.

## 1. Introduction

Machine learning (ML) models have been shown to predict the presence of clinical factors from medical imaging with remarkable accuracy [1,2]. ML models can achieve superior predictive performance with the use of algorithms that capture complex relationships between features, which can result in “black box” models that are difficult to interpret [3]. These methods use patient MR images as inputs and output a patient’s predicted probability of an outcome, without providing insight into how this prediction was derived. Thus, while these methods can outperform traditional prediction models, a potential barrier to use in clinical decision-making is a lack of transparency of how these predictions are arrived at.

To gain insight into these machine learning “black box” models and understand what factors contribute to a model’s prediction, there is tremendous interest in the use of explainable machine learning (XML) methods. This area was developed based on initial proposals by Ribeiro et al. [4] and Lundberg and Lee [5]. A recent overview of explainable machine learning methods was given in Covert et al. [6], demonstrating how various explanation approaches are related to each other. Some authors have criticized the fundamental premise of explainable machine learning methods that make post-hoc explanations rather than developing interpretable models [3,7]. However, many have taken advantage of the flexibility of XML methods and have applied them in various settings [8,9,10,11].

Explainable machine learning (XML) methods provide a way to obtain explanations from machine learning models that allow us to understand the relationships and importance of variables that are used in arriving at a particular prediction [5,12]. Model-agnostic explainer methods are independent of the ML algorithm used to develop the prediction model. Thus, once a best-fitting ML prediction model is identified, these explainer methods can be used to approximate and quantify the importance of each variable included in the predictive model. Some common model-agnostic explanation methods include Local Interpretable Model-agnostic Explanations (LIME) [4] and SHapley Additive exPlanations (SHAP) [5]. While the details of how each of these methods work differ, they generally behave by perturbing the input features to the model and observing the changes in the prediction. These explainers estimate the contribution of each feature included in the prediction model for a particular prediction. Use of an explainer method provides interpretability of black-box prediction models, which can reveal whether and how the algorithm uses clinically relevant information in its prediction. Thus, XML methods can increase clinician trust in how a model arrived at a particular prediction, and are increasingly being implemented when developing clinical prediction models using ML.

In recent years, analysis of medical images has become more quantitative through use of technologies such as radiomics, a method for automated high-throughput extraction of hundreds of quantitative features from medical images [13]. This process has the capability of uncovering anatomies that are difficult to see with the human eye and has the added benefit of creating a more reproducible analysis. Combined with ML methods, radiomics can be used to construct prediction models that are able to accurately predict clinical outcomes in biomedical research [14,15,16,17].

Prediction models using imaging data are commonly built using deep learning approaches that learn to recognize certain patterns within images based on their association with an outcome of interest, often through model architectures such as convolutional neural networks [18]. While these learned patterns contribute to a flexible approach to building predictive models from images, they are much harder to interpret due to their complexity [19]. Recent works on explainable deep learning-based imaging models commonly use methods such as integrated gradients and saliency maps to highlight areas of an image which are influential to a given model’s prediction [20,21,22,23,24]. Similarly, SHAP has been used to explain deep-learning imaging models [5,25] to the extent of highlighting the important areas of an image. While these methods can be useful for ensuring that the model is using information from sensible regions of the image (e.g., a tumor in cancer imaging) and not from random artifacts, it can be difficult to interpret what it is about that region that is important. 

Radiomics allows for the extraction of quantitative radiomics features, which fall into one of three categories: First-order features, which include summary measures (e.g., mean and median) of voxel intensities, second-order features which describe the distribution of voxel intensities in space (e.g., textures), and third-order features, which summarize the shape of a region of interest (ROI) [26]. After extraction, these features can be analyzed for association with some outcome of interest or included as features in prediction models. Previous literature has demonstrated that the extracted radiomic features and not just the image can be important in clinical decision-making [13,27]. In contrast to the image data utilized in deep learning approaches, radiomics features are well-defined, thus when included in prediction models allow for greater interpretability. The application of XML methods, e.g., LIME [28] and SHAP [29], to explain predictions from models built using radiomics features is still sparse, but would provide a powerful tool for identifying which features contribute to an explanation and how. 

Thus, while there are existing methods for developing prediction models with radiomics [30,31,32] and explaining ML models with XML tools such as LIME [4] and SHAP [5], these methods have not been commonly used in an integrated way for imaging data to explain predictions from models built with radiomic features. In this manuscript, we present a novel pipeline for the development of an interactive image analysis tool that can accept unprocessed medical images and perform the necessary steps of model development and explainer method application to make predictions and extract insight from images.

This paper contributes to the existing literature in XML methods for radiomics by demonstrating how to build and explain black-box prediction models using quantitative radiomics features. The presented approach supports a wide variety of ML prediction algorithms (e.g., penalized regression, random forests, and neural networks) in building a prediction model, and uses a model-agnostic explainer to show how the ML algorithm is using radiomics features to arrive at a particular prediction. Instead of just identifying areas on an image as being important, the proposed methodology can answer hypotheses related to whether tumor shape, image intensity, and texture are contributing to a patient’s prediction for use in clinical decision making. To our knowledge, much of the research in explainable machine learning has focused on the computational side [6]. In practice, many studies now report explainable machine learning outputs, whereas our goal is to suggest that this interpretation should occur in an interactive visualization framework.

To demonstrate this tool, we use retrospective study data of patients with glioma, a common type of cancer that develops in the glial cells of the brain and accounts for more than 70% of all brain tumors [33]. There is interest in developing a prediction model using MRI since mutations of the isocitrate dehydrogenase (IDH) gene have been shown to be a marker of oncogenesis and is one of the most specific biomarkers in the diagnostic classification of secondary glioblastoma multiforme [34]. We apply our proposed pipeline and visualization to this data set to present an explainable prediction model for IDH mutations using radiomics data that can be used as a tool in clinical decision-making. 

## 2. Materials and Methods

**Figure 1:**
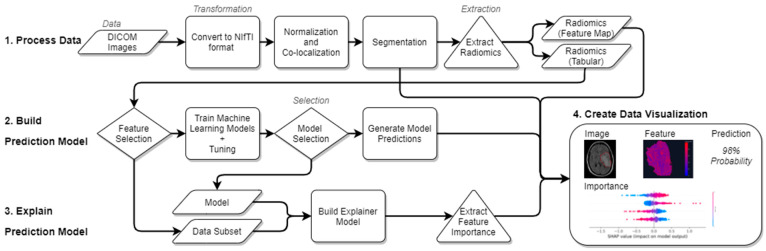
Methods Overview. Information enters the pipeline as DICOM images and then proceeds sequentially through the “Process Data”, “Build Prediction Model”, and “Explain Prediction Model” sub-pipelines, while select outputs are combined into a visual dashboard.

In this proposal, we explore the implementation of machine learning interpretability in the context of medical imaging to improve transparency of model decision-making. Our approach, visualized in Figure 1, builds on the workflow outlined by Lambin et al. [13], and can be generalized in the following steps:

1. *Imaging*. Images need to be captured and processed for consistency. Details of this step will vary by imaging modality, but common elements of post-processing include standardizing voxel intensity and co-registering multiple imaging sequences such that subject anatomy exists within the same volume as images from other sequences.
2. *Segmentation.* It may be desirable to limit the analysis of an image to some region of interest (ROI) within the image. Segmentation can be achieved by manually defining a bounded volume within the image with the help of software such as 3D Slicer, or either fully or semi-autonomously extracted using deep learning models e.g., V-nets [35].
3. *Feature Extraction*. Radiomics features are extracted from the ROI and outputted in one of two formats: Tabular data, where each feature has a numeric measurement for each image, and Feature maps, which visualize radiomics measures in the space of the ROI.
4. *Model Training and Evaluation*. Radiomics features are used as predictors of the clinical outcome of interest in a ML model. Any ML model for tabular data can be used here. Common model choices include elastic nets, gradient boosting machines, or support vector machines. Common model training workflows include testing many modeling algorithms, tuning model parameters, and validating predictive performance on new data.
5. *Model Explanation*. The behavior of the predictive model is estimated using an explainer model (i.e., LIME or SHAP).
6. *Explanation Visualization.* Presenting a visualization of the explanation can help summarize the model behavior. Explanations can be shown at the subject-level by plotting the variable importance for all predictors for a given subject. Alternatively, cohort-level explanations can be explored by aggregating all subject-level explanations into a single plot.

For the final step, we have developed an interactive dashboard which provides an intuitive interface with which clinicians can generate and interpret machine learning predictions. 

## 3. Application

We demonstrate the use of our proposed framework using a publicly available data set of MRI containing glioma. We build an ML model using radiomics features to predict mutations of the isocitrate dehydrogenase NADP+ 1 (IDH1) gene, which has been shown to be a marker of oncogenesis and is one of the most specific biomarkers in the diagnostic classification of secondary glioblastoma multiforme [34]. We describe the process from image processing to model development and finally visualization for use as a tool in clinical decision-making.

### 3.1. Imaging

Retrospective patient, clinical, and imaging data was obtained using a TCGA-GBM Dataset [36], which was obtained from The Cancer Imaging Archive [37]. This data set contained MR images of adult diffuse gliomas (WHO grades II, III and IV) for 204 subjects where IDH mutation was known (84 Mutant, 120 Wild Type). The available MRI sequences for these patients were T2-weighted and fluid-attenuated inversion recovery (FLAIR). Images were available in the DICOM format and were converted to NIfTI format and processed for standardization as follows: T2-weighted images were resampled to 1 mm isovoxel resolution, and FLAIR images were registered to T2 images after skull stripping, using the FMRIB software library [38]. After image registration, image signal intensity was normalized using the WhiteStripe R package (v2.3.1) [39].

### 3.2. Segmentation

Entire tumor areas (defined as areas of T2 hyper-intense tumor and edema on FLAIR images) were segmented by using semi-automatic methods, including signal intensity thresholding, region growing, and edge detection, with an open-source software (Medical Image Processing, Analysis and Visualization, https://mipav.cit.nih.gov/ (accessed on 1 April 2020). When necessary, segmentations were manually corrected by a trained neuroradiologist collaborator (YSC).

### 3.3. Feature Extraction

Radiomic features were extracted from all segmented regions of interest (ROIs) using PyRadiomics [40] and are detailed in Table 1. As two images per subject were available, radiomics features were extracted from both images separately using the same set of features. Images were first processed using one of two filtering steps: Wavelet filtering, which applies high or low pass filters in each of the 3 dimensions of the image resulting in 8 unique filtering combinations, and Laplacian of Gaussian filtering which can be conceptually understood as an edge-enhancing filter and filtered at three different levels of detail. Radiomics features were then extracted from each of these filtering configurations–12 in total: 1 original image, 8 wavelet filters, and 3 Laplacian of Gaussian filters. Extracted features from each of these filtered images were consistent and belonged to one of several groups of features: first order features which are generally summary statistics of the abundances of voxel intensities (e.g., mean, median, skew), second order features which can be conceptually thought of as textures or distributions of signal intensities through space, and shape features which concern the 3-dimensional shape of the ROI. As some features were equivalent across image types and filters (e.g., shape features), redundant features were eliminated from the feature set. In total, 1046 features were used for further analysis. 

### 3.4. Model Training and Evaluation

Several common ML models were trained to demonstrate the model-agnostic properties of our approach: An Elastic Net [41])–a penalized logistic regression used as an example of a relatively low complexity model, A Random Forest [42] an ensemble of random decision trees and an example of a model with moderate complexity, and two popular implementations of boosted ensembles of decision trees: XGBoost [43], and LightGBM [44]. These machine learning models were used to predict the binary outcome of IDH mutation using features extracted from T2 images alone, FLAIR images alone, and both T2 and FLAIR images under three different features selection scenarios: 1. No feature selection (models were trained with all extracted features), 2. Features selected prior to model training using recurrent feature elimination [45] by least absolute shrinkage and selection operator (LASSO) [46], and 3. Features selected using recurrent feature elimination by Random Forest. Hyperparameters for each model were tuned using Hyperopt [47], a Bayesian optimization algorithm, which maximizes average k-fold cross-validated (where k = 5) receiver operating characteristic area under the curve (ROC-AUC) over 1000 iterations. Hyperparameter search spaces are provided in Appendix A. The best performing model was identified based on ROC-AUC.

### 3.5. Model Explanation

Explainer models for each machine learning model were developed using SHAP to compute feature importance rankings and prediction contributions for each subject. The SHAP framework is model-agnostic and estimates model behavior through perturbation of model inputs. The SHAP algorithm is derived from game theory and assigns each feature an importance value for each individual prediction, which can be used to explain which features most contributed to a given patient’s prediction. The Shapley value for a feature is computed as the weighted average of the difference between the prediction from a model trained with and without the feature over all possible feature subset combinations that do not include the feature [5]. Due to the computational complexity when dealing with many features, the Shapley values are approximated using a sampling procedure [5,48]. A positive (negative) SHAP value indicates an increase (decrease) in the prediction, and a value of zero indicates no contribution of that feature to an individual’s prediction. SHAP values are unique, consistent, and locally accurate additive attribution values [5], where the total sum of SHAP values across all of a patient’s features equals their prediction, in this case, the probability of IDH mutation. Details on the SHAP algorithm and how to compute SHAP values can be found in [5]. To assess the overall magnitude of influence of each variable on model predictions within this cohort, SHAP values were aggregated as follows:

$$\mathrm{mean}\;{\left|\mathrm{SHAP}\right|}_{i}=\frac{\sum_{j=1}^{n}\left|{\mathrm{SHAP}}_{ij}\right|}{n}$$

where *i* denotes a feature, *j* denotes a subject, and *n* is the number of subjects. |SHAP*_ij_*| values range from 0 to 1, where higher values indicate greater importance of predictor *j* to patient *i*’s prediction. The mean |SHAP| provides an overall explanation of how important the feature is in the overall cohort, with higher values indicating greater overall importance.

### 3.6. Explanation Visualization

A data visualization dashboard was created using the python programming language and the Dash application framework [49]. The visualization was designed to show subject-level explanations from out top performing predictive model. Components in the visualization include:

1. *Image*. The MRI can be viewed and navigated using included controls for selecting the sequence (T2 or FLAIR), the view (Axial, Sagittal, or Coronal), and the slice (2D slices within a 3D image). The region of interest (ROI) can be toggled on or off and is overlayed on the image. A button to jump to the highest cross-sectional area slice of the ROI is also present.
2. *Prediction.* The probability of IDH mutation from the ML model, expressed as a percentage.
3. *Feature Map.* A visual map of radiomics features in the space of the ROI. A drop-down menu of extracted features can be used to explore maps of all extracted features.
4. *Subject Feature Importance.* A bar plot of the feature importances for the selected subject ordered by magnitude of importance and colored by the subject’s value relative to the rest of the cohort. Negative SHAP values correspond with an associated decrease in IDH mutation probability while positive SHAP values correspond with an increase in probability. Hovering over each bar will show the precise SHAP and feature values for the selected subject, and clicking a given feature bar will change the feature map to that feature and the image to the corresponding MRI sequence (e.g., T2-weighted, FLAIR).
5. *Model Feature Importance*. A bar plot of mean |SHAP| values for each feature in the cohort across all subjects. This plot provides a summary of the overall most important features within the cohort as a reference when viewing importances for a selected subject. Similarly to the subject feature importance plot, clicking a bar will change the displays of the feature map and image to match.
6. *Model Feature Influence*. A scatter plot of the SHAP values by feature value. This plot provides a visualization of how SHAP values change with respect to the underlying feature measure. For linear models, this will appear as a slope, but may be non-linear for more complex models.

## 4. Results

### 4.1. ML Model Performance

**Figure 2:**
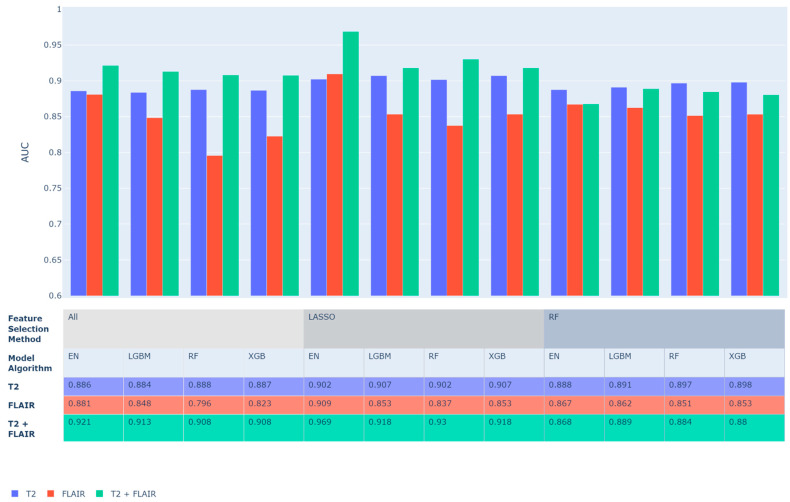
ML models five-fold cross-validated AUC predictive performance. LASSO and Random Forest (RF) indicate the method that was used to perform feature selection while All is used to indicate models with no feature selection (i.e., uses all features).

Predictive performance for all models was computed and ranked by five-fold cross-validated AUC. Figure 2 shows the best performing model from each family of prediction algorithms. In general, many methods performed well, with an average AUC of 0.88 (SD = 0.03) and many models achieving AUC values greater than 0.9. Using features from T2 alone led to significantly better predictive performance compared to using featured from FLAIR alone (*p* = 0.0002) with an average improvement in AUC of 0.042. Marginal improvements in AUC were achieved using both T2- and FLAIR-extracted features with an average improvement of 0.01 over T2 alone (not significant). Models with feature selection by LASSO performed better than model without feature selection (*p* = 0.0001) or with feature selection by Random Forest (*p* = 0.026). The top performing model by AUC was an Elastic Net with features selected prior to training by LASSO, which achieved a cross-validated AUC of 0.969 using both T2 and FLAIR images for each subject.

### 4.2. Model Explanations

**Figure 3:**
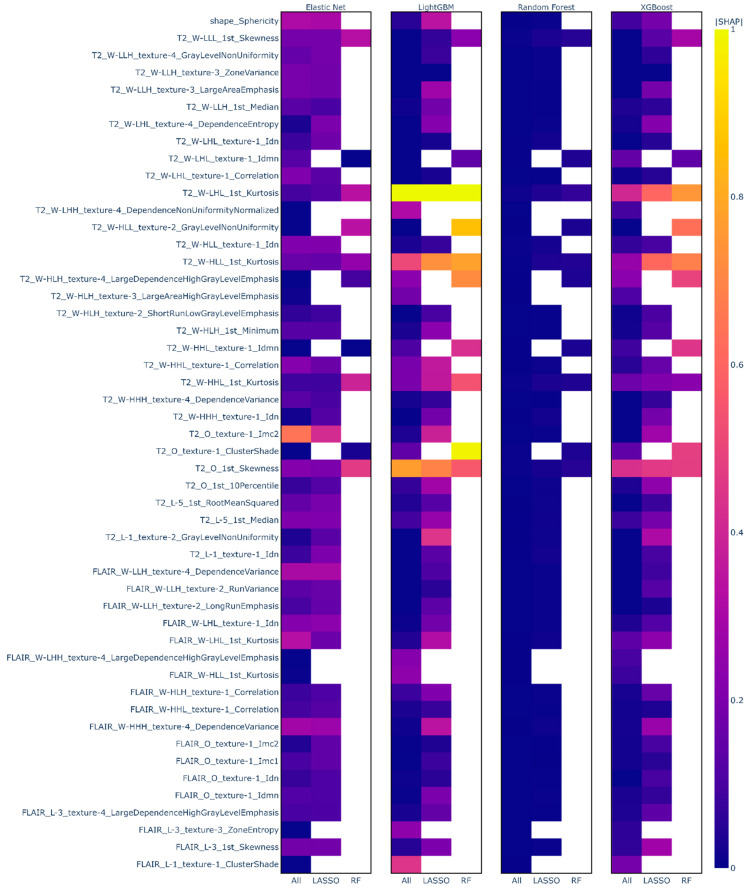
Heatmaps of mean |SHAP| for features by model. Top 50 features by sum of |SHAP| value across all models, sorted by feature similarity. Variable importance is shown by model family and feature subset, either All Features (All), selected by LASSO (LASSO), or Random Forest (RF) recurrent feature elimination. The highest mean |SHAP| values are shown in yellow while lower values are shown in blue on a continuous scale shown to the right. White cells indicate that the feature was not selected for that model.

In Figure 3, |SHAP| values are presented for individual features by model and feature selection method, where higher values indicate higher importance in the model. For Elastic Net models, we see that most features have |SHAP| values between 0.1 and 0.6, indicating a low to medium level of importance in the model. In these models, there is no single feature that is highly important (|SHAP| > 0.8), rather there are many somewhat important features. This is in contrast with both gradient boosted decision tree models (XGBoost/LightGBM), which show a small number of highly important radiomics variables, namely first order Kurtosis and Skewness, along with many low and medium level importance features. In Random Forest models, it appears as though all features have a low level of importance. This issue is beyond the scope of the current manuscript and will be explored in future research.

**Figure 4:**
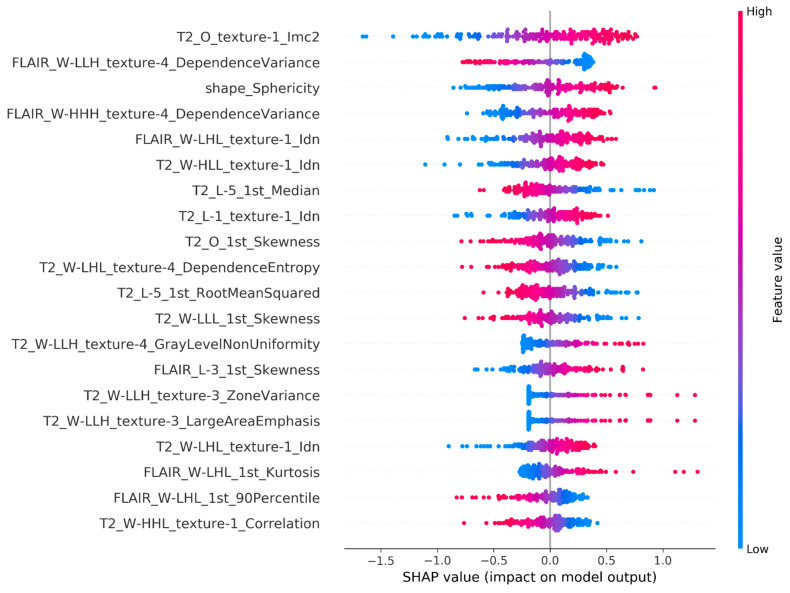
The top 20 radiomics features identified by SHAP for the top Elastic Net model ordered from most to least important. Positive SHAP values are associated with an increase in probability of mutation while negative values with a decrease in probability. Features with higher magnitudes of SHAP values have a greater effect on the model prediction. Each point represents a subject-level SHAP value. Points are colored by their relative value to the overall cohort with high values being shown in red and low values in blue.

In Figure 4, we present the prediction explanations for the LASSO-selected Elastic Net model, which was selected as the best model by AUC. This plot provides an explanation of how the radiomics features affect individual patient predictions as well as the cohort by demonstrating feature importance and directionality of association. The ranking of the features from top to bottom indicates the most to least important features, as measured by mean |SHAP|. The top 20 most important features include first-, second-, and third-order features extracted from both T2-weighted and FLAIR images. The two strongest predictors of IDH mutation are T2_O_texture_1-Imc2 and FLAIR_W-LLH_texture-4_DependenceVariance. From previously published work, combining visual inspection of T2-weighted and FLAIR images for developing glioma diagnostic models has performed well in the clinic [50], and has been validated with computational quantitative analysis [51]. Our results provide indirect support of this finding and lay the foundation for further explainability of these radiomic features and clinical translation. Additionally, several of the top predictors are related to morphology and intensity profile, which have been identified to be the most reproducible radiomics features [52], again suggesting robustness of our results.

The directionality of how a feature is associated with the prediction is assessed by the horizontal location (SHAP value) and the coloring of the dots, which identifies the feature value (red high, blue low). For example, for T2_O_texture_1-Imc2 we can see that high values of this feature (red dots) are associated with a higher predicted probability of IDH mutation (positive SHAP value). By plotting the SHAP values of a feature for all the individuals in the cohort, we can identify outlier effects. For example, FLAIR_W-LHL_1st_Kurtosis is not the most important feature globally in the cohort, but it is the most important feature for some individuals. This plot also shows that there are many distinct SHAP values for some features, such as shape_Sphericity, indicating that the importance of the feature varies for different individuals. 

### 4.3. Interactive Visualization

**Figure 5:**
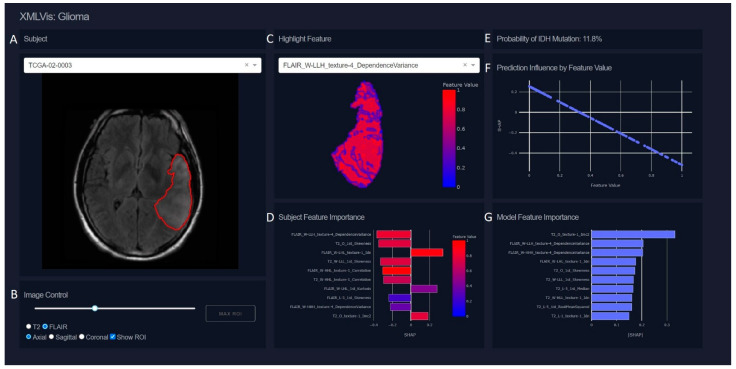
Screenshot of an interactive visualization tool for individual patient model prediction explanations. (**A**) The T2 MRI is shown of the axial slice with the highest cross-sectional area of the ROI (denoted by red boundary). (**B**) Image control options allow navigating through slices via a slide bar, toggling images type between T2 and FLAIR, changing view angle between Axial, Sagittal, and Coronal views, and toggling the ROI display. (**C**) Voxel-wise measurements of this feature are displayed in the space of the ROI. Here, FLAIR_W-LLH_texture-4_DependenceVariance is shown with a gradient color scale where high values are displayed in red, and low values in blue. (**D**) Bar plot of top 10 SHAP values for this patient’s model prediction. Features are ranked by |SHAP| and are colored by the average value of that feature as it relates to the entire cohort; colors on this scale are linked to colors in (**C**). SHAP values can be either positive -corresponding to a higher probability of IDH mutation–or negative–corresponding to a lower probability. The magnitude of the SHAP value denotes the overall importance of that variable to the prediction. (**E**) The predicted probability of IDH mutation for this patient was 11.6%. (**F**) A reference plot for how values of the selected feature (FLAIR_W-LLH_texture-4_DependenceVariance) affect the probability of IDH mutation. High values of FLAIR_W-LLH_texture-4_DependenceVariance are associated with lower SHAP values, indicating the patient is less likely to be IDH mutated with higher levels of FLAIR_W-LLH_texture-4_DependenceVariance. (**G**) Mean |SHAP| for all subjects in the cohort.

To demonstrate a patient-specific explanation of our model, we developed a web-based interactive visualization tool, guided by feedback from trained neuroradiologists (RJ, YSC). A demo with 5 example subjects is available from https://bit.ly/3pDcn5F (accessed on 1 April 2020). It allows users to view prediction explanations and images for individual patients in the cohort. Figure 5 shows a screenshot of the tool where the MRI are displayed along with the predicted probability of IDH mutation and plots which display information about the importance of features in the prediction. The SHAP values for the top 10 features for a given subject are displayed as a bar plot which can be clicked to display a feature map of the selected feature within the ROI. While average values for each feature are used in the model predictions, the feature map provides an intuitive way to understand what the feature represents in a medium familiar to visually inclined neuroradiologists. A plot displaying the top 10 features for the entire cohort is also shown for reference and can be clicked to show a feature map for the selected patient and feature. After using the tool, our radiologist collaborators provided the feedback that interacting with model explanations and seeing a visual representation of radiomics features helped to conceptualize how the predictive model was working.

## 5. Discussion

We have presented and demonstrated a novel pipeline for how machine learning can use radiomic features along with a combination of explainable machine learning (XML) and data visualization methods to predict outcomes from medical images in a way that gives insight into the model’s prediction process. Explanations as to which features contributed most to a patient-specific prediction can be measured and aggregated to understand the feature’s importance in a patient cohort. Together, these techniques improve interpretability of black-box algorithms without sacrificing the predictive performance and could help improve and expedite their acceptance in clinical practice for use in medical decision-making. 

One strength of our proposed pipeline is the use of a model-agnostic explainer approach. Due to the independence of the predictive and explainer models, XML can be applied to any model of any level of complexity to produce model explanations. The best-performing predictive model in our application was an Elastic Net model, which could be explained without the use of XML methods since it is conceptually similar to a linear regression model. However, if a less interpretable ML model (e.g., XGBoost) was identified as best-performing during the model development stage in the pipeline, the process is generalizable such that it can still be explained using the same metric (i.e., SHAP values as in Figure 3). Additionally, while we have demonstrated the application of our pipeline using SHAP values that we chose based on their increasing popularity for explaining clinical prediction models, alternative implementation can use other explanation methods (e.g., LIME) and associated visualizations within the same framework as indicated by the field of research or user preference. 

Another advantage of this approach is the development of ML models using well-defined radiomics predictors, instead of using deep-learning methods to merely identify image areas that are of high importance. Some of the radiomic features identified as important predictors of IDH mutation may not be familiar to the clinical radiologists because these features are usually not part of the standard clinical radiology lexicon. However, implementation of XML can help expedite ML use in clinical practice by allowing a more visual and explainable correlation with standard imaging features seen on MRI. Having knowledge that these patterns are important predictors could also inspire further studies into the biological basis for these patterns, leading to greater understanding of glioma biology. 

The visualization dashboard serves as a proof-of-concept for an automated MRI analysis tool based on radiomic features. The compilation of images, prediction probabilities, and important features into a cohesive patient prediction report provides a comprehensive view of a patient’s medical prognosis. In future work, we will improve the dashboard utility to radiologists and evaluate its effectiveness applying current methods in the field of Human-Computer Interaction [53]. While we demonstrated how such a tool could work, we plan to continue development to be able to handle the end-to-end process of performing this analysis with any dataset including features such as image preprocessing and automated tumor segmentation. Such a comprehensive tool would reduce the technical barriers for radiomics analyses experienced by many medical practitioners. 

Use of the methodology in this paper may be appropriate in any setting when there is a need to understand the image features which influence a predictive model. While we focused on MRI in this paper, radiomics features can be extracted from many other imaging modalities such as computed tomography (CT) and positron emission tomography (PET). This pipeline can be used in a variety of settings to obtain explanations from models that use a set of features to predict a clinical outcome of interest. It is important to note however that the quality of a model explanation is dependent on the quality of the model itself. The explainer model can only explain the prediction model, not the true generative process that the predictive model attempts to estimate. An explanation of a model with poor predictive accuracy should logically produce a poor-quality explanation. Similarly, the generalizability of the predictive model influences the generalizability of the explanations. Care should be taken to ensure that predictive models are properly validated before model explanations are given credence.

Limitations of this work include the difficulty in empirically comparing this methodology to existing methods, as there are not established metrics for what constitutes a good explanation when the underlying generative process that the prediction model is estimating is unknown. Future work in establishing relevant metrics for explanation quality is desirable. As the goal of this work is to improve trust in predictive models, feedback from clinicians will be essential to establishing sensible metrics. A qualitative study assessing the clinician’s trust and usability of saliency maps compared to or combined with SHAP feature importances of radiomic features may identify the direction in which future explainability research for medical imaging should be directed.

## 6. Conclusions

In conclusion, we have proposed a data processing pipeline to create an automated image analysis tool which uses methods of explainable machine learning to produce a patient-specific explanation for a model prediction. In contrast to methods for explainability of deep learning imaging models, such as integrated gradients that highlight areas of importance in an image, our method allows one to discern what it is about that area that was important. Our approach seeks to provide physicians with state-of-the-art predictive tools while giving them methods for validating model behavior to improve trust in predictive models.

## Figures and Tables

**Table 1:** Radiomic feature naming scheme and high-level overview.

[Image]_[Filter]_[Feature Group]_[Feature Name]		
Position	Abbreviation	Description
Image	T2	T2-weighted MRI. The sequence weighting highlights differences in the T2 relaxation time of tissues.
	FLAIR	Fluid-Attenuated Inversion Recovery is a special inversion recovery sequence with a long inversion time. This removes signal from the cerebrospinal fluid in the resulting images 1. Brain tissue on FLAIR images appears similar to T2 weighted images with grey matter brighter than white matter but CSF is dark instead of bright.
Filter	O	Original image. No additional filters are used to extract these features.
	W	Wavelet filtering yields 8 decompositions per level (all possible combinations of applying either a High (H) or a Low (L) pass filter in each of the three dimensions. Wavelet feature names are accompanied by a 3-letter sequence representing these filters.
	L	Laplacian of Gaussian filter, edge enhancement filter. Emphasizes areas of gray level change, where sigma (1, 3, or 5) defines how coarse the emphasized texture should be. A low sigma emphasis on fine textures (change over a short distance), where a high sigma value emphasizes coarse textures (gray level change over a large distance).
Feature Group	1st	First-order statistics describe the distribution of voxel intensities within the image region. (19 features)
	texture1 *	Gray level co-occurrence matrix (GLCM). (22 features)
	texture2 *	Gray level size zone matrix (GLSZM). (16 features)
	texture3 *	Gray level run length matrix (GLRLM). (16 features)
	texture4 *	Gray level dependence matrix (GLDM). (14 features)
	shape	3-dimensional shapes. (10 features)
Feature Name		Measured feature from feature group. See pyRadiomics documentation for feature descriptions.

* Texture featured renamed from their original name assigned by pyRadiomics for ease of interpretation. Detailed feature explanations available from: https://pyradiomics.readthedocs.io/en/latest/features.html# (accessed on 1 April 2020).

## Data Availability

Data from The Cancer Genome Atlas Glioblastoma Multiforme (TCGA-GBM) collection are available from https://wiki.cancerimagingarchive.net/display/Public/TCGA-GBM (accessed on 1 April 2020).

## Appendix A

**Table A1:** Hyperparameter Search Space for Prediction Model Training.

Algorithm	Parameter	Search Space
XGBoost	eta	Uniform (0.001–0.2)
	gamma	Uniform (0, 1)
	max_depth	Integers (3, 10)
	min_child_weight	Uniform (1, 10)
	subsample	Uniform (0.5, 1)
	colsample_by_tree	Uniform (0.5, 1)
	lambda	Uniform (0, 0.2)
	alpha	Uniform (0, 2)
LightGBM	num_leaves	Integers (30, 1000)
	learning_rate	Uniform (0.01, 0.2)
	min_child_samples	Integers (1, 10)
	reg_alpha	Uniform (0.1, 0.2)
	reg_lambda	Uniform (0.1, 0.9)
	subsample	Uniform (0.5, 1)
Random Forest	max_depth	Integers (3, 20)
	n_estimators	Integers (100, 1000)
Elastic Net	l1_ratio	Uniform (0, 1)
